# Deoxycorticosterone Producing Tumor as a Cause of Resistant Hypertension

**DOI:** 10.1155/2010/372719

**Published:** 2010-06-30

**Authors:** Saurabh Gupta, Jose Melendez, Apurv Khanna

**Affiliations:** ^1^University of Connecticut Health Center, Farmington, CT 06030, USA; ^2^SUNY Upstate Medical University, Syracuse, NY 13210, USA

## Abstract

We describe a young woman with longstanding resistant hypertension. Evaluation for renal artery stenosis and primary aldosteronism was unrevealing. In this setting of a suppressed plasma aldosterone concentration (PAC) and a suppressed plasma renin activity (PRA), a differential diagnosis of a deoxycorticosterone (DOC) producing tumor was entertained. Biochemical and imaging studies confirmed this diagnosis. Rare and novel DOC producing tumors are an important cause of resistant hypertension.

## 1. Introduction

Hypertension is a ubiquitous health problem. The overall worldwide prevalence of hypertension in the adult population is around 26% [1]. A significant proportion of these individuals have hypertension that is difficult to control with conventional treatment. Such patients are more likely to have an underlying identifiable cause of hypertension [2]. Identification of these causes is therefore acquiring greater therapeutic importance for the clinician.

## 2. Case Presentation

A 35-year-old African American female presented to the University Clinic for evaluation of resistant hypertension. She had onset of hypertension at the age of 17 years and since then had suboptimally controlled blood pressure despite the use of 4 antihypertensive medications including a diuretic. When first seen in the clinic, she was 3 weeks post partum from delivery of a preterm infant due to preeclampsia. Her only complaint in the office was that of fatigue which was present even before pregnancy. Family history was positive for hypertension in her mother and maternal grandfather, developing in middle age. Medications included Lisinopril 20 mg daily, Hydrochlorthiazide 25 mg daily, Amlodipine 10 mg daily, and Doxazosin 1 mg at bedtime.

On examination, her blood pressure was 150/90 mmHg with a heart rate was 88. Physical examination was unremarkable other than trace pedal edema. There were no cushingoid features or virilisation noted on exam. Pertinent laboratory values included a serum sodium of 135 (137–144 meq/L), serum potassium 3.1 (3.6–5.1 meq/L), serum bicarbonate of 26 (22–26 meq/L), blood urea nitrogen 9 (8–24 mg/dL), and serum creatinine of 0.8 (0.6–1.2 mg/dL). At this first visit the patient received oral potassium supplementation, Lisinopril dose was increased to 40 mg daily and Amiloride 5 mg twice daily was added to the regimen. Despite these measures patient continued to have poorly controlled blood pressure.

Prior to referral to us, for evaluation of uncontrolled hypertension accompanied by hypokalemia in a young female, Magnetic Resonance Angiography (MRA) of the renal arteries to look for fibromuscular dysplasia was ordered by her treating physician. The MRA did not show any evidence of renal artery stenosis. The MRA also did not show any adrenal mass, though this investigation was done primarily to screen for a vascular lesion and would not define a mass as well as a conventional MRI. As we will highlight in the following sections, based on the results of her biochemical studies, this diagnosis was in fact, less likely and this would not have been our initial screening test. On evaluation by us, to screen for primary aldosteronism, plasma aldosterone concentration (PAC), and plasma renin activity (PRA) levels were ordered. The PAC was suppressed at <4.0 ng/dL and the PRA level was also suppressed at <0.6 ng/mL/hr (0.6–4.0). In view of the suppressed PAC and PRA levels, the patient underwent a 24-hour urine collection for free cortisol and a plasma deoxycorticosterone (DOC) level was checked. The 24-hour urine collection was significant for an elevated free cortisol level of 219 (<45.0 mcg/day). The plasma deoxycorticosterone (DOC) levels were also markedly elevated at 65 (1.6–5.6 ng/dL). This leads us to suspect the presence of a cortisol and deoxycorticosterone cosecreting adrenal tumor. Subsequently a CT scan of the abdomen with intravenous contrast was done. It showed a 5.3 cm × 4.5 cm × 7 cm mass inferior to the right adrenal gland. The patient underwent a laparoscopic partial right adrenalectomy.

**Figure 1:**
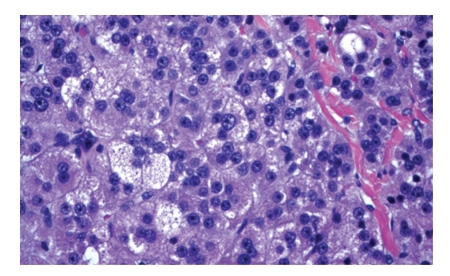
Adrenal cortical adenoma demonstrating focal high grade nuclear atypia.

**Figure 2:**
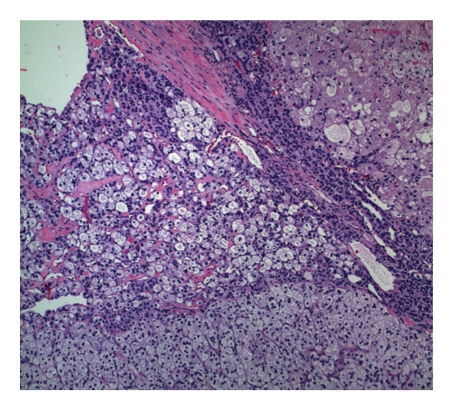
Adrenal cortical adenoma with areas of focal necrosis.

Histology revealed a right adrenal cortical adenoma on frozen section. While there was focal high grade nuclear atypia and focal necrosis, the findings were below the threshold for malignancy (see Figures 1 and 2). Three months postresection, patient had normalization of DOC and urine free cortisol levels. She also exhibited marked improvement in blood pressure control and was tapered off all her hypertensive medications. Her blood pressure was recorded at 100/50 mmHg at followup.

## 3. Discussion

Our approach towards patients with resistant hypertension involves obtaining a plasma renin activity (PRA) and a plasma aldosterone concentration (PAC) as an initial step. This helps separate patients into one of three categories:

(1) Those with a high PAC  :  PRA ratio; (2) those with a high PAC and high PRA, and (3) those with a low PAC and PRA levels. This is based on a high PAC  :  PRA ratio defined as >20 [3], and a high PAC value of ≥16 ng/dL [4].

**(1) High PAC : PRA Ratio**

The principal diagnosis here is of primary aldosteronism. The high PAC  :  PRA ratio is a good screening test but it is not diagnostic of this disorder. Further confirmation is provided by the aldosterone suppression test. Patients are salt-loaded either by mouth for 3 days or intravenously. The 24-hour urinary sodium excretion should be >200 mEq to document adequate sodium repletion. Urinary aldosterone excretion >12 mg/24 hours is consistent with hyperaldosteronism [3]. Once confirmed, patients need further testing with selective adrenal venous sampling for aldosterone to distinguish unilateral aldosterone producing adenomas (APA) from bilateral idiopathic hyperaldosteronism (IHA) [5]. The treatment of IHA is pharmacologic with use of mineralocorticoid receptor blockers while APA is treated surgically with unilateral total adrenalectomy.

**(2) High PAC : High PRA**

Patients presenting with a high PAC accompanied by a high PRA are further worked up for the presence of a unilateral renal artery stenosis or more rarely for a renin-secreting tumor. Renal arteriography remains the gold standard for diagnosis renal artery stenosis. Patients with fibromuscular disease respond better to angioplasty than those with atherosclerotic disease [6]. Renin secreting tumors are rare entities arising from the juxtaglomerular cells and are detected by radiologic imaging of the abdomen [7].

**(3) Low PAC : Low PRA**

The presence of a low PAC accompanied by a low PRA, as was present in our patient leads to differential diagnosis of bilateral renal artery stenosis, Cushing's syndrome, congenital adrenal hyperplasia (CAH), apparent mineralocorticoid excess (AME), deoxycorticosterone (DOC) producing tumors, and Liddle's syndrome. Cushing's syndrome can be caused either by excess endogenous cortisol or iatrogenically by excess steroid administration. A urinary-free cortisol level four times greater than normal is diagnostic of this disorder [8]. Apparent mineralocorticoid excess (AME) is a rare disorder causing a severe form of early onset hypertension. It is transmitted as an autosomal recessive trait. Patients suffer from a deficiency of 11*β*-hydroxysteroid dehydrogenase type 2 enzyme. This enzyme normally converts the more active cortisol to the inactive cortisone. In conditions of enzyme deficiency, cortisol continues to flood the mineralocorticoid receptor causing sodium retention, potassium wasting, and hypertension. This syndrome is commonly found in settings of familial consanguinity and there is extensive target organ damage, nephrocalcinosis, and renal failure [9]. Our patient did not have these clinical features including consanguinity and renal failure. If urine cortisol levels are not elevated, then as the next step, we check serum deoxycorticosterone (DOC) levels. If elevated, as was present in this case, the differential is that of CAH versus a DOC producing tumor. CAH is most commonly caused due to 21 hydroxylase deficiency and this is not associated with hypertension. CAH due to deficiency of 11*β*-hydroxylase is seen most in the Middle East and is characterized by virilization of the infant and 17-hydroxylase enzyme deficiency is often manifest at birth and is also mainly seen in pediatric populations. However, partial enzyme deficiencies have been observed in hirsute women [10]. Thus in adult patients, congenital enzymatic defects are less likely compared to the likelihood of a DOC-producing tumor. This is investigated further with imaging of the abdomen. In the literature, DOC-producing tumors have been described only in a handful of cases [11]. In our patient, CT scan of the abdomen did indeed reveal an adrenal mass. Finally, if all testing is negative, the diagnosis of Liddle's syndrome is entertained. These patients have a mutation of the *β* or *γ* subunit of the renal epithelial sodium channel, causing is increased sodium reabsorption in the distal nephron [12]. This leads to manifestations similar to mineralocorticoid excess, such as hypertension, hypokalemia, and metabolic alkalosis. However, plasma renin activity and plasma aldosterone concentrations are secondarily suppressed in these patients. Treatment consists of blocking distal nephron sodium channels with drugs like amiloride and triamterene. In conclusion, in patients with resistant hypertension accompanied by low PRA and low PAC, Cushing's syndrome is an important diagnosis to be considered. If, however, Cushing's syndrome is ruled out, then in adult patients, rare deoxycorticosterone producing tumors are a distinct possibility.
